# A First Report on Multidrug-Resistant *Escherichia coli* O25 ST131 Dissemination in an Outpatient Population in Zagreb, Croatia

**DOI:** 10.3390/antibiotics14020109

**Published:** 2025-01-21

**Authors:** Maja Anušić, Tatjana Marijan, Ana Mlinarić Džepina, Vladimira Tičić, Lucija Gršković, Jasmina Vraneš

**Affiliations:** 1 Department of Clinical Microbiology, Dr. Andrija Štampar Teaching Institute of Public Health, 10000 Zagreb, Croatia; tatjana.marijan@stampar.hr (T.M.); anamldz@gmail.com (A.M.D.); vladimira.ticic@stampar.hr (V.T.); jasmina.vranes@stampar.hr (J.V.); 2 Department of Medical Microbiology and Parasitology, School of Medicine, University of Zagreb, 10000 Zagreb, Croatia; lucijapp@gmail.com

**Keywords:** *Escherichia coli* O25 ST131, urinary tract infections, molecular epidemiology, multidrug-resistant clone, ESBL production, fluoroquinolone resistance

## Abstract

Background/Objectives: Antimicrobial resistance of the *E. coli* O25 ST131 clonal lineage poses a significant therapeutic challenge worldwide, often involving resistance to fluoroquinolones and extended-spectrum beta-lactamase (ESBL) production. This retrospective study compared the dissemination of multidrug-resistant *E. coli* O25 ST131 isolated from the urine of outpatients at the largest Croatian clinical microbiology department across six years over two study periods. Methods: The *E. coli* O25 ST131 clonal lineage was detected via a rapid PCR method using *pabB* and *trpA* primers after positive agglutination with *E. coli* serogroup O25 antisera. ESBL phenotypes and antibiotic susceptibility were investigated according to EUCAST guidelines and breakpoint tables. Results: In the first period, there were a total of 45 isolates of *E. coli* O25 ST131, among which 30 were isolates with proven ESBL production. In the second period, a total of 114 isolates of *E. coli* O25 ST131 were detected, among which 75 (65.8%) were ESBL-positive (*p* > 0.05). In ESBL-negative strains, the multidrug-resistant (MDR) phenotype was characterized by simultaneous resistance to ampicillin, co-trimoxazole, and fluoroquinolones (with an equal proportion of 3/15 isolates in the first period and 7/39 isolates in the second period, *p* > 0.05). There was no statistically significant difference in the frequency of MDR detection across the two study periods (36/45 and 98/114, *p* > 0.05). This is the first detection of *E. coli* O25 ST131 in the outpatient population in Zagreb. Conclusions: There was no statistically significant difference in the frequency of detecting the *E. coli* O25 ST 131 clone across the two study periods. The high frequency of MDR phenotype among ESBL-negative isolates of *E. coli* O25 ST131 and an equally high proportion of MDR strains among ESBL producers in this clonal lineage, with the total detection of MDR isolates ≥ 80% in both study periods, are the reasons why this bacterial clone poses a public health threat and why further investigation into its metabolic and virulence characteristics is needed in order to estimate its spreading potential among the outpatient population in Zagreb.

## 1. Introduction

Uropathogenic *E. coli* (UPEC) plays an essential role in causing urinary tract infections. Worldwide, an increment in its resistance rates to antimicrobial drugs has been observed, especially to beta-lactams and fluoroquinolones which are used as first-line treatments for urinary tract infections. The occurrence of multidrug-resistant (MDR) phenotypes of UPEC leads to difficulties in choosing an appropriate antibiotic treatment and is often the cause of antibiotic therapeutic failure. The *E. coli* O25 ST131 clonal lineage of the phylogenetic group B2 is recognized as a pandemic and multidrug-resistant clone, and up to 50% of extended-spectrum beta-lactamase (ESBL)-positive or fluoroquinolone-resistant *E. coli* strains belong to this clonal lineage [1,2]. According to its antimicrobial resistance profiles and bacterial population genomes, *E. coli* ST131 is divided phylogenetically into three major clades (A, B, and C). The evolution of fluoroquinolone resistance in the *E. coli* O25 ST131 clonal lineage occurs within clade C, which has the gene *fim*H30 that encodes a type 1 fimbrial adhesion structure on the cell membrane. High-level resistance to fluoroquinolones is due to two chromosomal mutations in the genes *gyrA* and *parC.* Fluoroquinolone-resistant *E. coli* O25 ST131 classified in clade C is named CH30R [3]. Most isolates of *E. coli* O25 ST131 belong to this CH30R clade. The origin of clade C was most likely from North America around 1980 [4,5]. Over time, *E. coli* O25 ST131 clade C also acquired resistance to beta-lactams. This resistance is mainly due to the production of CTX-M beta-lactamases. *E. coli* O25 ST131 subclade C1 usually presents CTX-M14 and CTX-M27 beta-lactamase production, while subclade C2 presents CTX-M15 beta-lactamase production. The *E. coli* O25 ST131 clonal lineage with the ESBL phenotype and fluoroquinolone resistance is defined as clade CH30Rx.

*E. coli* O25 ST131 is found in humans, animals, and the environment, but humans remain its main reservoir. The first studies on the intercontinental presence of this clonal lineage, which was proven in an international collection of *E. coli* isolates from Europe, Asia, and North America, date back to 2008 [6]. In Europe, ciprofloxacin-resistant *E. coli* O25 ST131 caused community-acquired uncomplicated cystitis between 2003 and 2006 [7]. *E. coli* isolates causing UTIs and bacteriaemia were reported to predominantly belong to the *E. coli* O25 ST131 clonal linage in the northwest of England during the years 2004 and 2005 [8]. Multidrug-resistant *E. coli* O25 ST131 was a major cause of serious infections in the United States of America during the year 2007 [9]. The first reported detection of *E. coli* ST131 in Croatia was at the Clinical Hospital Center in Zagreb during the year 2005. The isolate was ciprofloxacin-resistant and ESBL-positive [10]. The fast dissemination of this pandemic clone was attributable to its metabolic properties and capability of acquiring virulence and resistance genes [11]. In 2020, the first report of the ST131 clone in an outpatient setting in Croatia was published, which was detected using whole-genome sequencing in six isolates from the Dubrovnik-Neretva County [12].

Data about the spread of this MDR clone in the outpatient population in Zagreb are missing. Due to the aging population in Zagreb and the spread of MDR strains from hospitals to the community, it is expected to find similar prevalence of the O25 ST131 clone as in other European countries. The aim of this study was to report the prevalence of uropathogenic *E. coli* O25 ST131 among fluoroquinolone-resistant *E. coli* strains in the outpatient population in Zagreb over two study periods (from March 2011 to January 2012 and from October 2017 to August 2018), and to compare the rates of MDR phenotypes and ESBL production of the strains collected in those two periods.

## 2. Results

### 2.1. Prevalence of E. coli O25 ST131 Clonal Lineage

During the two study periods, the laboratory for urinary tract infections received 29,544 and 52,193 midstream urine samples for urine culture in the first and second study periods, respectively. Uropathogenic *E. coli* was isolated from 4354 and 7717 specimens in the first and second periods, respectively, but only 151 and 368 O25 fluoroquinolone-resistant strains isolated with a count ≥10^5^ CFU/mL were included in this study. The prevalence rates of *E. coli* serogroup O25 were 47/151 (31.1%) and 122/368 (33.1%); our results show a slightly higher prevalence than a study conducted in Mexico by Paniagua-Contreras G.L. et al., which reported a prevalence of 20.6% for serogroup O25 in patients with community-acquired UTIs during 2013, and a study conducted the same year in Iran by Momtaz H. et al., who reported a prevalence of 26% in patients with symptomatic UTIs [13,14]. The reasons for the higher prevalence in our study could be due to the inclusion criteria, such as resistance to fluoroquinolones.

**Figure 1:**
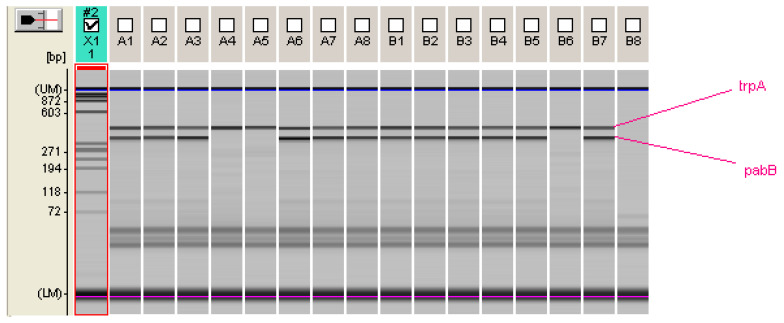
Microchip electrophoresis for *pabB* and *trpA* genes in *E. coli* O25 ST131. trpA—427 bp, pabB—347 bp; DNA ladder-X1; positive isolates *E. coli* O25 ST131—A1, A2, A3, A6, A7, A8, B1, B2, B3, B4, B5; negative isolates *E. coli* O25—A4, A5; *E. coli* ATCC 25,922—B6; positive control *E. coli* ST131—B7; negative control H2O for PCR—B8.

Further characterization of the collected strains with the rapid PCR method described by Clermont et al. for the detection of the *E. coli* ST131 clonal lineage by using the two primers *pabB* and *trpA* showed 45 and 114 positive fluoroquinolone-resistant *E. coli* O25 ST131 strains in the two study periods, respectively (Figure 1) [15]. A total share of 95.7% and 93.4% of the investigated O25 bacterial strains belonged to the *E. coli* O25 ST131 clonal line. These results are very similar to the results of a previous surveillance study on *E. coli* isolated from blood, which was conducted from 2011 to 2017, where 93.3% of the *E. coli* serogroup O25 belonged to the *E. coli* ST131 clonal line [16]. 

The prevalence of fluoroquinolone-resistant *E. coli* O25 ST131 detected in the two study periods was 29.8% and 30.9%, respectively. This is the first report of uropathogenic *E. coli* O25 ST131 prevalence in the outpatient population in Zagreb.

### 2.2. Antibiotic Susceptibility of E. coli O25 ST131

**Figure 2:**
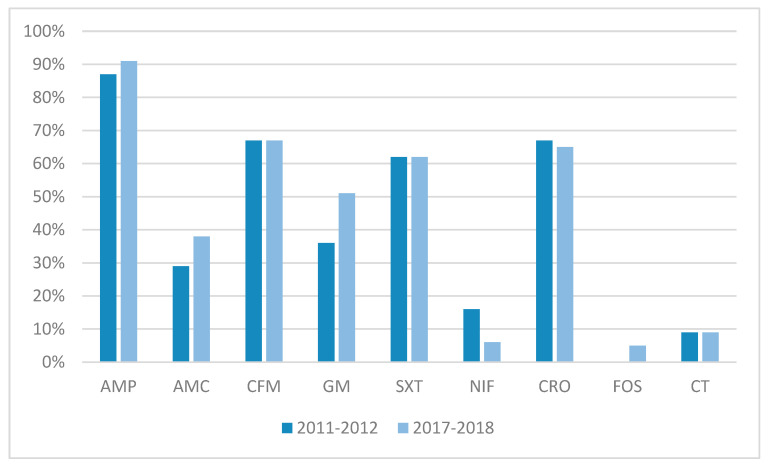
Comparison of resistance to antibiotics in two study periods; AMP–ampicillin, AMC–amoxicillin clavulanic acid, CFM–cefixime, GM–gentamicin, SXT–sulfamethoxazole–trimethoprim, NIF–nitrofurantoin, CRO–ceftriaxone, FOS–fosfomycin, CT–ceftolozane–tazobactam.

The susceptibility of 159 fluoroquinolone-resistant isolates of *E. coli* O25 ST131 to 19 antibiotics, tested according to EUCAST breakpoints, is shown in Table 1 and Figure 2.

In terms of susceptibility to antibiotics, the uropathogenic isolates of *E. coli* O25 ST131 in the outpatient population in Zagreb showed high resistance rates to antimicrobials, especially to aminopenicillin (86.7% and 91.2% in the first and second study periods, respectively), the first- (66.7% and 67.5%), second- (66.7%) and third-generation (66.7% and 64.9%) cephalosporins, and sulfamethoxazole/trimethoprim (62.2% in both study periods). The antimicrobials that these isolates showed low rates of resistance to included nitrofurantoin (15.6% and 6.1% in the first and second study periods, respectively) and fosfomycin (5.3% in the second study period). Resistance to fosfomycin was not detected in the first study period. All isolates of *E. coli* O25 ST131 were susceptible to carbapenems and ceftazidime/avibactam. The total numbers of detected ESBL-positive strains among the tested fluoroquinolone-resistant isolates of *E. coli* that belonged to the *E. coli* O25 ST131 clonal lineage were 30/48 (62.5%) and 75/122 (61.5%) in first and second study periods, respectively, without significant difference in the frequency of ESBL producers across the two study periods (*p* > 0.05).

### 2.3. Multidrug-Resistant Profiles of E. coli O25 ST131 Isolates

Multidrug-resistant phenotypes (resistance to ≥3 antimicrobial categories) were observed in the *E. coli* O25 ST131 clonal lineage in 36/45 (80%) and 98/114 (86%) cases across the two study periods, without statistically significant difference (*p* > 0.05). Multidrug resistance to ampicillin, co-trimoxazole, and fluoroquinolones was present in 3/15 (20%) and 7/39 (17.9%) cases of ESBL-negative *E. coli* O25 ST131, also without statistically significant difference when comparing the first and second study periods. The multidrug-resistant profile results are presented in Table 2. The most frequent multidrug-resistant profile of ESBL-positive *E. coli* O25 ST131 was co-resistance to beta-lactams with or without inhibitors and the first, second, third, or fourth generation of cephalosporins, which was accompanied with resistance to sulfamethoxazole/trimethoprim and aminoglycosides. Overall, there were 32 different multidrug-resistant profiles present in the *E. coli* O25 ST131 clonal lineage.

### 2.4. Patients’ Characteristics

In the first study period, fluoroquinolone-resistant *E. coli* was detected in 124 female and 27 male patients (M/F = 1:4.6) aged from 11 to 95 years (ẍ = 69); in the second study period, it was detected in 288 female and 80 male patients (M/F = 1:3.6) aged from 9 to 98 years (ẍ = 72). Among them, the O25 ST131 clone was detected in thirty-nine female and six male patients aged from 14 to 95 years (ẍ = 69) and in eighty-three female and thirty-one male patients aged from 9 to 93 years (ẍ = 72) in the first and second study periods, respectively.

The preliminary results of this study were presented in a poster session at the 13th Croatian Congress of Clinical Microbiology and the 10th Croatian Congress on Infectious Diseases (4th CROCMID) held on 20–23 October 2023 in Šibenik, Croatia.

## 3. Discussion

The ECDC antimicrobial surveillance reports for the years 2011, 2012, 2017, and 2018 estimated an increase in resistance of *E. coli* to fluoroquinolones ranging from 10% to 25% over the seven-year period in the majority of European countries, although this finding could be affected by EUCAST introducing revised clinical breakpoints for fluoroquinolones for all *Enterobacteriaceae* after 2016, leading to the higher fluoroquinolone resistance rate. Croatian national antimicrobial surveillance reports for the years 2011, 2012, 2017, and 2018 showed an increase in resistance rates of *E. coli* to fluoroquinolones ranging from 13% to 20%, which was similar to the ECDC reports for other EU countries [17,18,19,20]. Even though there was a significant difference between the rates of resistance to fluoroquinolones among the isolates of *E. coli* identified in the laboratory for urinary tract infections (13.2% and 21.1% in the first and second study periods, respectively; unpublished data), no significant difference in the prevalence of the fluoroquinolone-resistant *E. coli* O25 ST131 clonal line was observed over the seven-year study period. Around one-third of fluoroquinolone-resistant *E. coli* isolates belonged to this clonal lineage in our study, which was in accordance with the report about the emergence of *E. coli* O25 ST131 among ciprofloxacin-resistant *E. coli* isolates in Europe during the period from 2003 to 2006. Worldwide, the prevalence rates of uropathogenic *E. coli* O25 ST131 are different, but comparable prevalence rates to ours were found in studies conducted in the United Kingdom, Spain, Sweden, Canada, the USA, and Australia. The prevalence of uropathogenic *E. coli* O25 ST131 was 12.3% (37/300) in community and hospital urine isolates in Northwest England during 2007 and 2009 [21]. In Nottingham during 2008 and 2009, 22% of the isolates identified in outpatient and hospital urine samples of elderly patients belonged to the *E. coli* ST131 clonal lineage [22]. In the city of Lugo, Spain, the prevalence of *E. coli* O25 ST131 was 23.1% in 2006, 22.5% in 2007, and 20% in 2008 for *E. coli* ESBL-positive isolates identified mostly from the urine samples of outpatients and hospitalized patients [23]. In the city of Seville, the prevalence of *E. coli* ST131 was 12.3% in *E. coli* isolates and 23.5% in *E. coli* ESBL-positive isolates in 2010, where these *E. coli* isolates were mostly isolated (81.9%) from urine samples [24]. At the national level in Sweden during the years 2007, 2009, and 2011, a total of 236 *E. coli* ESBL-positive isolates were identified from urine samples in 2007, 304 isolates in 2009, and 463 isolates in 2011, which were tested using the polymerase chain reaction method with the primers for *pabB* to prove the presence of *E. coli* ST131. The prevalence of *E. coli* ESBL ST131 was 34–38%, and no significant increase was recorded during the observation period, with the clonal lineage of *E. coli* ESBL-positive ST131 being dominant over other sequence types of *E. coli* [25]. In Sweden during the years 2017 and 2018, the prevalence of E. coli ESBL ST131, as a cause of sporadic urinary tract infections, was 36% (83/229) in patients aged ≥ 15 years [26]. In Canada from 2002 to 2004, 46 (23%) isolates out of a total of 199 uropathogenic *E. coli* isolates belonged to *E. coli* ST131, with 96% of strains being resistant to fluoroquinolones [27]. In the USA from 2007 to 2010, a study identified a total of 94 ESBL-positive *E. coli* isolates and 158 *E. coli* isolates without extended-spectrum beta-lactamase production, which were mostly isolated from urine samples in an outpatient population, and the prevalence of *E. coli* ESBL ST131 was 50% (47/94) while that of isolates without ESBL production was 13% (21/158) [28]. In a 2011 study in the USA with two months of follow-up, among the *E. coli* isolates isolated mostly from urine samples, the prevalence of *E. coli* ST131 was 15% (27/180) in outpatients, 49% (48/98) in hospitalized patients, and 76% (28/37) in users of long-term care homes [29]. In Australia from 2007 to 2009, 582 *E. coli* isolates resistant to fluoroquinolones were isolated from clinical samples, mostly from urine samples of outpatients and hospitalized patients, and 202 (35%) of the isolates belonged to *E. coli* ST131 [30]. These studies and our study showed that the *E. coli* O25 ST131 clonal lineage is strongly associated with resistance to fluoroquinolones and ESBL production.

Studies on the resistance of the *E. coli* O25 ST131 clonal lineage to antimicrobials are diverse with respect to the inclusion criteria, such as the type of clinical specimens (urine, blood, or other sources of specimens), age and gender of patients, sample size and the source of infection (community- versus nosocomial-acquired infection), and observed time periods [31,32,33,34]. The clonal lineage of uropathogenic fluoroquinolone-resistant *E. coli* O25 ST131 in the outpatient population in Zagreb demonstrated resistance rates higher than 25% for 11 out of 19 antibiotics, ranging from 28.9% to 91.2%. The resistance rates to ampicillin/amoxicillin were 86.7% and 91.2% in the first and second study periods, respectively, which were similar to the rates reported in a study by Banerjee et al., in which the resistance rates to ampicillin were 98% in *E. coli* ST131 subclone H30 and 78% in *E. coli* ST131 non-H30 subclone isolated from community-associated urinary tract infections between 2007 and 2009 [35]. In Europe, comparable resistance rates to ampicillin (100% and 80%) were reported by Brisse et al. in clinical isolates of *E. coli* ST131 CTX-M producers and *E. coli* ST131 non-ESBL producers between 2008 and 2009 [36]. In a retrospective study by Olesen et al., among the 128 *E. coli* ST131 isolates collected from 1968 to 2011, resistance rates to ampicillin were 63% in *E. coli* ST131 non-H30 and 96% in *E. coli* ST131 H30 Rx [37]. The resistance rates to amoxicillin/clavulanic acid and piperacillin/tazobactam in our study were 28.9%/37.7% and 44.4%/32.5%, respectively. The study by Olesen et al. reported a resistance rate of 41% to amoxicillin/clavulanic acid in isolates of *E*. *coli* ST131, and similar resistance rates to amoxicillin/clavulanic acid (41%) and piperacillin/tazobactam (44%) were also reported by Peirano et al. between 2000 to 2010 [38,39]. In the USA, Kanamori et al. reported a resistance rate of 41% to piperacillin/tazobactam in isolates of *E. coli* ST131 between 2010 and 2015 [40]. The resistance rates to the first, second, and third generations of cephalosporins in our study were higher than 50% due to the high proportion of ESBL-positive isolates. The proportion of ESBL-producing fluoroquinolone-resistant *E. coli* O25 ST131 was 30/151 (19.8%) and 75/368 (20.4%) in the first and second study periods, respectively, while the proportion of ESBL-producing *E. coli* was 4.8% and 7% in the total number of *E. coli* isolates during the observed periods. All *E. coli* O25 ST131 isolates were susceptible to carbapenems and ceftazidime/avibactam. The low resistance rates of 15.6% and 6.1% to nitrofurantoin were comparable to the rates of 10% and 9% reported by Kudinha et al. and Rasouinasab et al., respectively [41,42]. The low resistance rates of 0% and 5.3% to fosfomycin were also comparable to the zero-resistance rate found in isolates of *E. coli* ST131 CTX-M producers in the study by Brisse et al., and the 4% resistance rate reported for isolates of *E.coli* ST131 H30 in an older patient group by Tchesnokova et al. [36,43]. The antimicrobial drugs nitrofurantoin and fosfomycin could be used as empirical treatment for uncomplicated cystitis against *E. coli* O25 ST131. The antimicrobial treatment for complicated urinary tract infections against *E. coli* O25 ST131 ESBL should be the use of carbapenems. At present, the E. coli O25 ST131 clonal lineage has developed resistance to carbapenems and colistin, which further complicates the antimicrobial therapeutic options.

*E. coli* O25 ST131 is strongly associated with multidrug resistance, especially if it presents a pattern of ESBL production and fluoroquinolone resistance [37,44,45]. In this study, co-resistance to aminoglycosides and sulfamethoxazole/trimethoprim in fluoroquinolone-resistant *E. coli* O25 ST131 that produces ESBL was the most frequent multidrug-resistant profile, and the same finding was observed in the study by Olesen et al. [38]. Antimicrobial multidrug resistance is a major issue in public health, which often causes failure in empirical treatment for urinary tract infections and could significantly increase the risk of morbidity and mortality.

The limitation of this retrospective study is a lack of medical data about the types of urinary tract infections (uncomplicated or complicated), prescribed antimicrobial therapy, or previous hospitalization of the patients. The age and sex of the patients were the only available demographic characteristics at the time of analysis and, due to the old age of most patients with detected O25 ST131 *E. coli* infection, it could only be speculated that these patients were frequently hospitalized because of co-morbidity and were exposed to antibiotics.

## 4. Conclusions

In summary, this study showed no difference in the prevalence of fluoroquinolone-resistant *E. coli* O25 ST131 across the two study periods, despite the observed significant difference in the rates of resistance to fluoroquinolones in UPEC in Zagreb across the seven-year period. There was also no statistically significant difference in the proportion of multidrug-resistant isolates among the *E. coli* O25 ST131 strains identified in the two study periods, with a similar frequency of MDR (around 80%) and ESBL production rate (around 60%) in both periods. Further investigation into the virulence and metabolic characteristics of the strains collected in these two periods and of the recently collected isolates is needed to estimate the spreading potential of this MDR clone in the outpatient population in Zagreb.

## 5. Materials and Methods

### 5.1. Bacterial Isolates

Bacterial isolates of *E. coli* resistant to fluoroquinolones were isolated from urine culture in two time periods, with the first period between March 2011 and January 2012 and the second period from October 2017 to August 2018. Monoculture of nonduplicated consecutive *E. coli* isolates was included if leucocyte esterase and a bacterial count ≥10^5^ CFU/mL were detected in the outpatients’ samples of midstream urine received at the laboratory for urinary tract infections at Andrija Štampar Teaching Institute of Public Health, Zagreb, Croatia. The isolates of *E. coli* were then preserved in semi-solid agar at room temperature and at −80 °C in commercial cryoprotective media (Microbank, Pro-Lab Diagnostics) for further testing.

### 5.2. Strains Identification Analysis

The urine samples were inoculated with 10 µL sterile loops on to blood and MacConkey agars (Oxoid Ltd., Altrincham, England). *E. coli* species identification was confirmed by using standard biochemical tests (glucose/lactose fermentation, indole production, negative citrate and phenylalanine utilization, positive lysine and/or ornithine decarboxylation) The GN biochemical identification card for Vitek 2 compact instrument (bioMerieux, Marcy-l´Etoile, France) was also performed for all *E. coli* O25 ST131 isolates giving excellent and very good identification results.

### 5.3. Detection of E. coli O25 ST131 Clonal Lineage

All isolates were agglutinated with *E. coli* serogroup O25 antisera (Denka Seiken Co.) according to the manufacturer’s protocol. Detection of the clonal group *E. coli* ST131 was performed on all positive isolates of the *E. coli* serogroup O25 using the rapid PCR method described by Clermont et al. with the primers O25pabBF: 5′-TCCAGCAGGTGCTGGATCGT-′3, O25pabBR: 5′-GCGAAATTTTTCGCCGTACTGT-′3, trpA.F: 5′-GCTACGAATCTCTGTTTGCC-′3, and trpA2.R: 5′-GCAACGCGGCCTGGCGGAAG-′3 [15]. Briefly, PCR was carried out in a 20 µL volume containing 2 µL of 10× buffer (supplied with Taq polymerase, Invitrogen, Thermo Fisher Scientific Inc., Waltham, MA, USA), 2 µL of the primer O25pabB F (20 pmol, Microsynth AG, Balgach, Switzerland), 2 µL of the primer O25pabB R (20 pmol, Microsynth), 1.2 µL of the primer trpA.F (12 pmol, Microsynth), 1.2 µL of the primer trpA2.R (12 pmol, Microsynth), 8 µL of dNTP (20 µM, Invitrogen, San Diego, CA, USA), 0.2 µL of *Taq* polymerase (1U, Invitrogen, San Diego, CA, USA), 0.6 µL of MgCl2 (5 mM), and 2.8 µL of bacterial lysate under the following PCR conditions: denaturation for 4 min at 94 °C, 30 cycles of 5 s each at 94 °C and 10 s each at 65 °C, and a final extension step of 5 min at 72 °C. The PCR products were analyzed via microchip electrophoresis using a MultiNa apparatus (Shimadzu), with *E*. *coli* ST131 proven by Multilocus sequence typing as a positive control and *E. coli* ATCC 25,922 as a negative control (Figure 1).

### 5.4. Antibiotic Susceptibility Testing

Susceptibility to antimicrobials was evaluated for the following antibiotics: ampicillin (10 µg), amoxicillin/clavulanic acid (20–10 µg), cephalexin (30 µg), cefuroxime (30 µg), cefixime (5 µg), gentamicin (10 µg), sulfamethoxazole/trimethoprim (23.75–1.25 µg), nitrofurantoin (100 µg), norfloxacin (10 µg), ciprofloxacin (5 µg), piperacillin/tazobactam (30–6 µg), ceftriaxone (30 µg), ceftazidime (10 µg), cefepime (30 µg), imipenem (10 µg), meropenem (10 µg), ertapenem (10 µg), amikacin (30 µg), fosfomycin (200 µg), ceftazidime/avibactam (10–4 µg), and ceftolozane/tazobactam (30–10 µg) via the disk diffusion method according to EUCAST v. 1.3 and EUCAST v. 7.1 breakpoint tables using antibiotic disks (Oxoid Ltd., UK and Mast Group Ltd., Bootle, UK).

### 5.5. Detection of ESBL Phenotype

The presence of the ESBL phenotype in the *E. coli* O25 ST131 isolates was detected following the recommended EUCAST methods: a combined disk test with antibiotic disks of cefotaxime, ceftazidime, ceftriaxone, and cefepime, either alone or in combination with clavulanic acid, and a double-disk synergy test with cefotaxime, ceftazidime, and cefepime next to a disk containing amoxicillin/clavulanic acid were performed [46]. An augmentation of the inhibition zone in the presence of clavulanic acid for at least 5 mm in the combined disk test or the distortion of the inhibition zones around the cephalosporin disk towards the central disk with clavulanic acid indicated the production of ESBL. *E. coli* ATCC 25,922 and *Klebsiella pneumoniae* 700,603 were used as the positive and negative control, respectively.

### 5.6. Statistical Analysis

χ^2^ tests were performed to assess statistical differences in the prevalence of strains during the study periods (Social Science Statistics), and a *p*-value < 0.05 was considered statistically significant.

## Figures and Tables

**Table 1:** Susceptibility of *E. coli* O25 ST131 clonal lineage to various antibiotics.

No	Antibiotic	2011–2012 Year No (%)			2017–2018 Year No (%)		
		S	I	R	S	I	R
1	Ampicillin/Amoxicillin	6 (1.3)	0	39 (86.7)	10 (8.8)	0	104 (91.2)
2	Amoxicillin-clavulanic acid	32 (71.1)	0	13 (28.9)	71 (62.3)	0	43 (37.7)
3	Piperacillin-tazobactam	25 (55.6)	0	20 (44.4)	77 (67.5)	0	37 (32.5)
4	Cefalexin	15 (33.3)	0	30 (66.7)	37 (32.5)	0	77 (67.5)
5	Cefuroxime	15 (33.3)	0	30 (66.7)	38 (33.3)	0	76 (66.7)
6	Cefixime	15 (33.3)	0	30 (66.7)	38 (33.3)	0	76 (66.7)
7	Ceftriaxone	15 (33.3)	0	30 (66.7)	38 (33.3)	2 (1.8)	74 (64.9)
8	Ceftazidime	20 (44.45)	5 (11.1)	20 (44.45)	39 (34.2)	6 (5.3)	69 (60.5)
9	Cefepime	20 (44.4)	7 (15.6)	18 (40)	40 (35.1)	17 (14.9)	57 (50)
10	Gentamicin	29 (64.4)	0	16 (35.6)	56 (49.1)	0	58 (50.9)
11	Amikacin	37 (82.2)	0	8 (17.8)	101 (88.6)	0	13 (11.4)
12	Sulfamethoxasole-trimethoprim	17 (37.8)	0	28 (62.2)	43 (37.8)	0	71 (62.2)
13	Nitrofurantoin	38 (84.4)	0	7 (15.6)	107 (93.9)	0	7 (6.1)
14	Fosfomycin	45 (100)	0	0	108 (94.7)	0	6 (5.3)
15	Imipenem	45 (100)	0	0	114 (100)	0	0
16	Meropenem	45 (100)	0	0	114 (100)	0	0
17	Ertapenem	45 (100)	0	0	114 (100)	0	0
18	Ceftazidime-avibactam	45 (100)	0	0	114 (100)	0	0
19	Ceftolozane-tazobactam	41 (91.1)	0	4 (8.9)	104 (91.2)	0	10 (8.8)

**Table 2:** Multidrug-resistant (MDR) profiles of *E. coli* O25 ST131 clonal lineage.

No.	MDR Profiles	1st Time Period 36 MDR Strains 2011–2012 No.	2nd Time Period 98 MDR Strains 2017–2018 No.
1.	AMP, AMC/PTZ, CIP	1	3
2.	AMP, AMC/PTZ, NIF, CIP	1	0
3.	AMP, SXT, CIP	3	7
4.	AMP, SXT, GM/AN, CIP	1	4
5.	AMP, AMC/PTZ, 1st CEF, SXT, CIP	0	1
6.	AMP, AMC/PTZ, SXT, FOS, CIP	0	1
7.	AMP, AMC/PTZ, SXT, CIP	0	3
8.	AMP, GM/AN, CIP	0	3
9.	SXT, NIF, CIP	0	1
10.	AMP; 1st, 2nd, 3rd or 4th CEF; CIP	1	3
11.	AMP; SXT; 1st, 2nd, 3rd or 4th CEF; CIP	4	9
12.	AMP; SXT; GM/AN; 1st, 2nd, 3rd or 4th CEF; CIP	6	15
13.	AMP; SXT; FOS; 1st, 2nd, 3rd or 4th CEF; CIP	0	1
14.	AMP; SXT; GM/AN; FOS; 1st, 2nd, 3rd or 4th CEF; CIP	0	1
15.	AMP; SXT; CT; 1st, 2nd, 3rd or 4th CEF; CIP	0	1
16.	AMP; SXT; GM/AN; CT; 1st, 2nd, 3rd or 4th CEF; CIP	0	1
17.	AMP; GM/AN; 1st, 2nd, 3rd or 4th CEF; CIP	2	7
18.	AMP; GM/AN; NIF; 1st, 2nd, 3rd or 4th CEF; CIP	1	1
19.	AMP; NIF; CT; 1st, 2nd, 3rd or 4th CEF; CIP	1	0
20.	AMP; AMC/PTZ; NIF; 1st, 2nd, 3rd or 4th CEF; CIP	0	1
21.	AMP; AMC/PTZ; 1st, 2nd, 3rd or 4th CEF; CIP	0	1
22.	AMP; AMC/PTZ; SXT; 1st, 2nd, 3rd or 4th CEF; CIP	4	5
23.	AMP; AMC/PTZ; SXT; CT; 1st, 2nd, 3rd or 4th CEF; CIP	1	0
24.	AMP; AMC/PTZ; SXT; GM/AN; 1st, 2nd, 3rd or 4th CEF; CIP	6	12
25.	AMP; AMC/PTZ; SXT; GM/AN; NIF; 1st, 2nd, 3rd or 4th CEF; CIP	2	1
26.	AMP; AMC/PTZ; SXT; NIF; CT; 1st, 2nd, 3rd or 4th CEF; CIP	0	1
27.	AMP; AMC/PTZ; SXT; GM/AN; CT; 1st, 2nd, 3rd or 4th CEF; CIP	1	4
28.	AMP; AMC/PTZ; SXT; GM/AN; FOS; 1st, 2nd, 3rd or 4th CEF; CIP	0	1
29.	AMP; AMC/PTZ; SXT; GM/AN; FOS; CT; 1st, 2nd, 3rd or 4th CEF; CIP	0	1
30.	AMP; AMC/PTZ; GM/AN; 1st, 2nd, 3rd or 4th CEF; CIP	0	7
31.	AMP; AMC/PTZ; GM; NIF; CT; 1st, 2nd, 3rd or 4th CEF; CIP	1	1
32.	AMP; AMC/PTZ; CT; 1st, 2nd, 3rd or 4th CEF; CIP	0	1

AMP: ampicillin; AMC: amoxicillin/clavulanic acid; PTZ: piperacillin/tazobactam; NIF: nitrofurantoin; SXT: sulfamethoxazole/trimethoprim; GM: gentamicin; AN: amikacin; 1st, 2nd, 3rd or 4th CEF: 1st, 2nd, 3rd, or 4th generation of cephalosporins; CT: ceftolozane/tazobactam; FOS: fosfomycin.

## Data Availability

The original contributions presented in this study are included in the article. Further inquiries can be directed to the corresponding author.
